# Localized, non-random differences in chromatin accessibility between homologous metaphase chromosomes

**DOI:** 10.1186/s13039-014-0070-y

**Published:** 2014-11-19

**Authors:** Wahab A Khan, Peter K Rogan, Joan HM Knoll

**Affiliations:** Department of Pathology and Laboratory Medicine, University of Western Ontario, London, ON N6A 5C1 Canada; Departments of Biochemistry and Computer Science, University of Western Ontario, London, ON N6A 5C1 Canada; Cytognomix, Inc, London, ON N6G 4X8 Canada

**Keywords:** Metaphase single copy FISH, Homologous chromosome structure, 3-D super resolution microscopy, Differential chromatin accessibility, Allelic differences, Human mitotic chromosomes, Molecular cytogenetics

## Abstract

**Background:**

Condensation differences along the lengths of homologous, mitotic metaphase chromosomes are well known. This study reports molecular cytogenetic data showing quantifiable localized differences in condensation between homologs that are related to differences in accessibility (DA) of associated DNA probe targets. Reproducible DA was observed for ~10% of locus-specific, short (1.5-5 kb) single copy DNA probes used in fluorescence in situ hybridization.

**Results:**

Fourteen probes (from chromosomes 1, 5, 9, 11, 15, 17, 22) targeting genic and intergenic regions were developed and hybridized to cells from 10 individuals with cytogenetically-distinguishable homologs. Differences in hybridization between homologs were non-random for 8 genomic regions (*RGS7*, *CACNA1B*, *GABRA5*, *SNRPN*, *HERC2*, *PMP22*:IVS3, *ADORA2B*:IVS1, *ACR*) and were not unique to known imprinted domains or specific chromosomes. DNA probes within *CCNB1*, *C9orf66*, *ADORA2B*:Promoter-Ex1, *PMP22*:IVS4-Ex 5, and intergenic region 1p36.3 showed no DA (equivalent accessibility), while *OPCML* showed unbiased DA. To pinpoint probe locations, we performed 3D-structured illumination microscopy (3D-SIM). This showed that genomic regions with DA had 3.3-fold greater volumetric, integrated probe intensities and broad distributions of probe depths along axial and lateral axes of the 2 homologs, compared to a low copy probe target (*NOMO1*) with equivalent accessibility. Genomic regions with equivalent accessibility were also enriched for epigenetic marks of open interphase chromatin (DNase I HS, H3K27Ac, H3K4me1) to a greater extent than regions with DA.

**Conclusions:**

This study provides evidence that DA is non-random and reproducible; it is locus specific, but not unique to known imprinted regions or specific chromosomes. Non-random DA was also shown to be heritable within a 2 generation family. DNA probe volume and depth measurements of hybridized metaphase chromosomes further show locus-specific chromatin accessibility differences by super-resolution 3D-SIM. Based on these data and the analysis of interphase epigenetic marks of genomic intervals with DA, we conclude that there are localized differences in compaction of homologs during mitotic metaphase and that these differences may arise during or preceding metaphase chromosome compaction. Our results suggest new directions for locus-specific structural analysis of metaphase chromosomes, motivated by the potential relationship of these findings to underlying epigenetic changes established during interphase.

**Electronic supplementary material:**

The online version of this article (doi:10.1186/s13039-014-0070-y) contains supplementary material, which is available to authorized users.

## Background

Homologous metaphase chromosome structures are heterogeneous at optical, sub-optical and atomic resolution [1–5]. This heterogeneity is manifest as distinctive chromosomal banding patterns superimposed on a highly conserved banding framework [6,7]. Within the same cell, each chromosome of a homologous pair may be laterally and longitudinally asymmetric [8,9] or display differences in DNA methylation [10], and replication timing [11–14]. Differences in chromosome band resolution and histone modifications are distributed along the length of the mitotic metaphase chromosomes [15]. In fact, phosphorylation of core histones-H3 and H4 at specific residues is retained in metaphase chromosomes, as an intermediate step in chromosome condensation [16]. By contrast, lysine methylation and acetylation of histones are transient chromosome marks, with the loss of acetylation observed on all core histones in G_2_/M-arrested cells [17,18]. High fidelity mitotic metaphase chromosome condensation is essential for accurate transmission and differentiation of the genome into daughter cells, however this process tolerates some degree of structural heterogeneity between chromosome homologs [1]. Despite advances in modeling higher order chromosome condensation, the locus-specific accessibility of chromatin within highly condensed metaphase chromosomes is not well understood. Some progress, however, has been made through investigations of histone and nonhistone proteins that reorganize chromatin into its condensed state [19].

We have noted reproducible differences in chromatin accessibility between homologous metaphase chromosomes in specific genomic regions using locus-specific short (1.5-5 kb), fluorescence in situ hybridization (FISH) probes [20,21]. These differences manifest as variation in hybridization intensities between homologs at single cell resolution. This phenomenon has been observed for ~10% of the 305 genomic probes that we have reported [20–25], however the reasons for such variation were not understood. The remaining genomic regions show no significant differences in hybridization intensities between allelic loci on metaphase chromosomes.

In this study, we investigated locus-specific targets in metaphase chromosome regions that show consistent differences in DNA probe fluorescence intensity between homologs. Evidence is presented that these differences in hybridization of DNA probes result from their differential accessibility (DA) to their respective genomic targets. Using optical, and super-resolution microscopy with short target, unique sequence single copy FISH probes; these allelic chromosome regions exhibit consistent, non-random differences between their respective chromosome structures. Further, sequence analyses of interphase epigenetic marks at these loci suggest the possibility that such differences may be related to the presence of specific chromatin modifications.

## Results

### Differential hybridization patterns detected on normal metaphase chromosome

**Figure 1:**
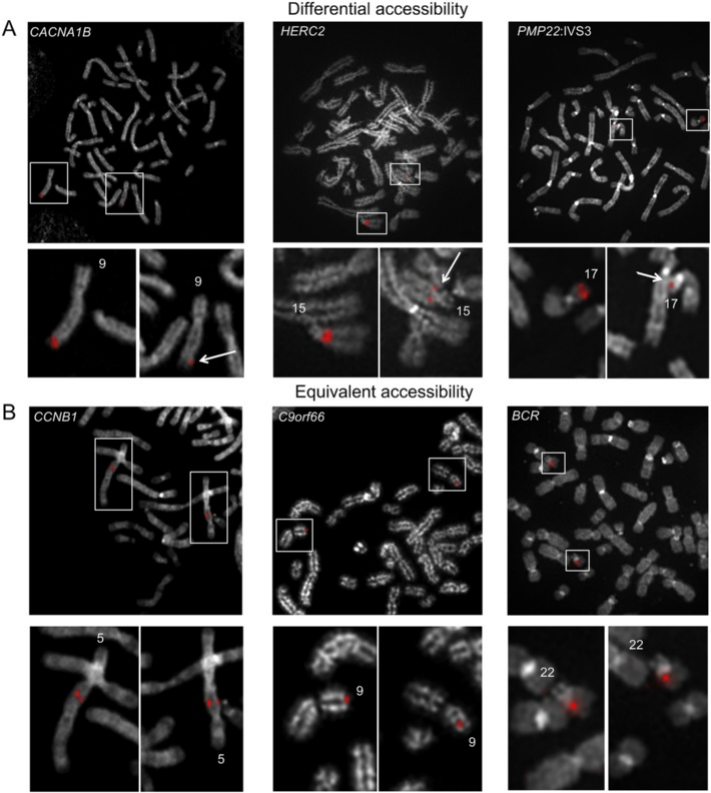
**Differential accessibility and equivalent accessibility patterns between metaphase chromosome homologs detected by single copy probes. A**. Human chromosomes hybridized with single copy FISH probes developed from *CACNA1B* (2.23 kb), *HERC2* (1.81 kb), and *PMP22:*IVS3 (2.32 kb) (left to right) show differential hybridization between homologs. Arrows indicate the homolog with less fluorescence (or less accessibility). **B**. Examples of human cells with single copy FISH probes developed from within *CCNB1* (2.47 kb)*, C9orf66* (2.08 kb), and *BCR* (3.4 kb) (left to right) that show similar fluorescence intensities (or equivalent accessibility) between homologous regions. Chromosomes were counterstained with DAPI (converted to gray scale in image) and probes were labelled with digoxigenin d-UTP and detected with Cy3 digoxin antibody.

Our previous studies demonstrated consistent differences in hybridization intensities for single copy probes in at least two-thirds of the metaphase cells. DA was probe and genomic interval specific and not related to either probe labeling or the individual samples hybridized. To illustrate different hybridization behaviours between homologs with short-target, single copy FISH probes, we compare examples of normal metaphase chromosomes hybridized with probes that show differences in accessibility to probes with equivalent accessibility. Single copy probes with differences in fluorescence intensities (i.e. differential accessibility or DA) between homologs (*CACNA1B*, *HERC2*, and *PMP22*:IVS3 genes) are shown in Figure 1A, Table 1 and are contrasted with hybridized probes that show similar fluorescence intensities (i.e. equivalent accessibility) to each homolog (*CCNB1*, *C9orf66*, *BCR*, Figure 1B and Table 1).

**Table 1:** **Comparison of open chromatin features to single copy genomic regions with and without DA**

		**Open chromatin features**					
**SC probe probe location [GRCh37]**	**Gene interval or cytoband**	**DNase-OS**	**FAIR-OS**	**H3K4me**	**H3K9Ac**	**H3K27Ac**	**H3K4me2**
chr1:1171789-1175143*	1p36.3, intergenic	11592	8710	544.0	168.4	115.6	120.6
chr1:1181574-1185503	*FAM132A*:Ex1-IVS1	7394	9695	596.5	143.0	102.8	160.9
chr1:1628792-1633615	*CDK11B*:IVS6	10378	14274	326.3	222.1	121.8	177.6
chr1:1632683-1637407	*CDK11B*:IVS6	12139	12829	290.7	125.8	51.8	51.8
**chr1:240965538-240967390**	*RGS7*:IVS13-IVS14	1420	9976	200.6	74.0	12.1	29.6
chr1:240988582-240990678*	*RGS7*:IVS4-IVS5	2840	8999	125.3	55.3	7.4	59.2
chr4:3242502-3246008	*HTT*:Ex67	9225	15222	248.0	142.8	81.4	51.8
**chr5:1421588-1425427**	*SLC6A3*:IVS4-IVS5	4702	8574	172.0	80.3	66.6	14.8
**chr5:9355970-9358454**	*SEMA5A*:IVS3	2827	16953	235.4	103.6	36.5	29.6
chr5:9361501-9365307	*SEMA5A*:IVS3	12398	40009	3017.6	597.4	1993.0	1530.2
**chr5:9371425-9374496**	*SEMA5A*:IVS3	1397	18058	531.5	48.5	81.4	59.2
chr5:11042187-11044508	*CTNND2*:IVS16	2221	15462	253.7	80.3	32.6	44.4
chr5:11071700-11076039	*CTNND2*:IVS16	4422	19382	344.8	158.4	118.5	77.8
chr5:11084988-11089067	*CTNND2*:IVS15	2942	16403	297.3	81.4	81.4	22.2
chr5:68462247-68464721*	*CCNB1*:Ex1-IVS3	31707	29162	1400.2	2378.9	1953.4	2076.3
chr7:73506616-73509661	*L1MK1*:IVS14	32349	30750	3473.0	4213.1	5870.8	4186.7
chr7:73534615-73536880	*L1MK1*:Ex1-IVS3	3639	9068	237.2	47.3	0	22.2
**chr8:116658428-116661455**	*TRPS1*:IVS1	3738	15369	650.0	224.2	78.6	396.1
**chr8:116661938-116665132**	*TRPS1*:IVS1	2031	15754	316.0	112.2	59.2	57.9
chr9:213762-215844*	*C9orf66*:Ex1	10945	15191	868.4	1550.5	503.2	1667.6
chr9:133587757-133589963	*ABL1*:Ex1b-IVS1b	32515	25514	616.8	3043.7	2278.6	1563.2
chr9:133616347-133618188	*ABL1*:IVS1b	1917	10733	341.9	83.6	42.0	71.1
chr9:133733132-133735051	*ABL1*:IVS3	2859	8103	188.8	50.9	74.2	37.0
chr9:133735369-133737639	*ABL1*:IVS3	2211	11425	259.7	62.8	56.1	95.6
chr9:133745513-133749828	*ABL1*:IVS4-IVS6	18884	37627	4959.4	477.6	982.4	998.7
chr9:133759487-133764440	*ABL1*:Ex11	7053	15356	322.9	174.4	142.9	74.0
chr9:140952206-140954439*	*CACNA1B*:Ex29-IVS31	4956	8277	302.2	88.8	33.8	51.8
**chr9:140969092-140971796**	*CACNA1B*:IVS33-IVS34	4127	7686	151.2	69.2	44.4	37.0
chr11:133180187-133182699*	*OPCML*:IVS1	2306	11280	202.1	77.4	37.0	133.8
chr12:11958559-11960434	*ETV6*:IVS2	2403	19900	2947.6	327.2	751.8	757.8
chr12:11992883-11994726	*ETV6*:IVS2	2257	16709	786.4	134.8	384.3	62.9
chr12:11992883-11995741	*ETV6*:IVS3	2988	23954	887.0	206.0	453.6	232.7
**chr13:100626271-100630715**	13q32.3, intergenic	11678	18628	216.6	76.2	65.8	23.2
**chr13:100643221-100648153**	13q32.3, intergenic	7452	25389	687.8	115.4	83.2	221.4
**chr15:22690247-22693115**	15q11.2, intergenic	1705	4996	347.0	85.2	47.4	29.6
**chr15:22853681-22855541**	*TUBGCP5*:IVS11-IVS13	1049	21106	132.0	75.4	96.9	22.2
chr15:23864038-23868139	15q11.2, intergenic	4260	17789	272.4	74.0	38.6	79.6
chr15:23883747-23886037	15q11.2, intergenic	1969	6908	84.0	76.3	10.5	29.6
chr15:23886989-23890525	*MAGEL2*:Promoter- 3′UTR	7602	12049	198.2	88.8	51.8	50.9
**chr15:25016909-25018586**	15q11.2, intergenic	1670	5764	216.6	38.2	48.1	111.5
**chr15:25052358-25054037**	15q11.2, intergenic	671	6051	83.8	51.9	7.4	0
chr15:25068481-25070727*	*SNRPN:*Promoter:IVS1	1524	7291	149.1	74.4	19.3	22.2
chr15:25199392-25201602	*SNRPN*:IVS4	6799	10253	258.1	1391.0	937.3	486.3
chr15:25613407-25617676	*UBE3A*:IVS7-IVS8	2728	26796	3025.0	81.4	182.8	96.0
chr15:27117096-27119866*	*GABRA5*:IVS3	5815	8082	140.0	40.8	37.0	29.6
chr15:28509526-28511337*	*HERC2*:IVS12-IVS13	4580	10908	854.2	313.8	728.2	465.3
**chr15:102388168-102389774**	*OR4F13P*:IVS3-Ex5	950	8872	64.0	47.6	37.0	29.6
chr16:15013674-15017156	16p13.11, intergenic	814	984	248.3	51.8	62.2	53.0
chr16:16412325-16415807	*PKD1P1*:IVS2-IVS7	473	268	168.0	76.8	71.4	51.8
chr16:16452359-16455837	16p13.11, intergenic	418	670	98.7	54.1	65.2	44.4
chr16:16234893-16236784	*ABCC1*:IVS30-Ex31	4867	11513	451.2	103.6	89.3	45.6
chr16:18440574-18444056	16p12.3, intergenic	110	0	181.5	74.0	73.4	31.2
chr16:18484058-18487536	16p12.3, intergenic	616	907	167.7	89.9	51.8	19.1
**chr17:905599-910582**	*ABR*:IVS21-3′UTR	10824	19481	304.9	174.9	132.8	2.9
chr17:941273-943865	*ABR*:IVS16	3508	6315	170.9	89.1	74.0	71.8
chr17:2591614-2594572	*CLUH*:IVS25-3′UTR	10756	11515	576.5	96.2	384.9	163.2
chr17:2596810-2599164	*CLUH*:IVS13-IVS19	5323	6551	301.9	112.5	63.3	37.0
chr17:2603297-2606091	*CLUH*:IVS3-IVS9	7600	7093	222.1	57.0	51.8	123.2
chr17:18128679-18133300	*LLGL1*:Promoter-Ex2	36213	27618	1016.0	647.8	219.2	743.9
chr17:18143933-18146387	*LLGL1*:Ex17-IVS22	7467	8690	136.8	114.2	37.0	16.8
chr17:15133018-15136902*	*PMP22*:IVS4-Ex5	5482	14180	207.3	113.3	60.8	34.6
chr17:15150757-15153084*	*PMP22*:IVS3	4694	12616	321.0	77.7	28.7	44.4
**chr17:15174803-15176657**	17p12, intergenic	2574	11	163.0	89.3	37.0	31.4
chr17:15847751-15849832*	*ADORA2B*:Promoter-Ex1	10751	9889	763.1	241.5	79.0	980.8
chr17:15868752-15870532*	*ADORA2B*:IVS1	2859	17457	711.4	95.0	78.8	84.7
chr17:18150509-18152632	*FLII*:IVS15-Ex21	23767	14999	2289.9	74.7	439.4	75.6
**chr17:18153505-18154823**	*FLII*:IVS12-IVS14	3547	3937	47.1	36.7	36.8	0
chr17:19286892-19288934	*MFAP4*:IVS3-Ex6	3415	5897	286.5	139.3	95.8	54.8
**chr17:37861465-37863632**	*ERBB2*:IVS5-IVS6	5114	6170	296.6	90.6	78.3	84.4
chr17:37882684-37886219	*ERBB2*:IVS27-Ex31	9666	16440	1561.2	617.8	331.8	836.4
chr17:38500482-38504359	*RARA*:IVS2	17458	19211	3584.2	597.6	650.9	813.2
**chr17:38512106-38514271**	*RARA:*IVS8-Ex9	5468	6830	177.2	130.6	125.8	88.3
chr17:38608442-38610468	*IGFBP4*:IVS1-IVS3	4526	10512	230.8	39.5	29.6	62.7
chr17:38613433-38617530	*IGFBP4*:Ex4	8748	18085	557.1	155.4	88.8	74.0
chr17:80290070-80293112	*SECTM1*:Ex1-IVS1	13714	13008	2005.4	294.1	202.4	1002.5
chr20:10642756-10644909	*JAG1*:IVS2-IVS3	2943	15006	1104.1	118.6	49.2	225.7
chr21:36259933-36264124	*RUNX1*:IVS2	26119	30777	1920.4	2050.4	1478.7	2915.8
chr21:39454065-39456057	*DSCR4*:IVS2	2440	7032	155.4	65.3	24.6	65.9
chr21:39463783-39466136	*DSCR4*:IVS2	2017	10359	126.5	63.2	51.8	25.6
chr21:39473031-39475467	*DSCR4*:IVS2	2256	10103	137.4	86.9	37.0	0.2
chr22:19338598-19342289	*HIRA*:IVS21-IVS24	3429	12123	325.1	150.0	59.2	37.0
chr22:23578368-23581572	*BCR*:IVS1	6792	19899	2489.1	283.0	240.2	284.8
chr22:23604414-23607814	*BCR*:IVS4	11921	14381	1989.6	425.4	621.7	362.3
chr22:23623055-23625566	*BCR*:IVS8	21132	16241	2543.9	311.6	501.8	451.4
chr22:51175125-51178674*	*ACR*:Ex1-IVS3	11986	12916	175.2	85.1	37.0	58.9
**chrX:592626-595515**	*SHOX*:IVS2-Ex3	7316	7254	125.9	55.3	29.6	37.0
**chrX:597816-600430**	*SHOX*:IVS3	6234	7821	64.0	74.0	41.5	44.4
**chrX:602538-605057**	*SHOX*:IVS5	4319	4191	147.9	29.6	7.0	14.8
chrX:7891853-7895877	*PNPLA4*:Ex1-IVS2	19932	42372	1854.3	2340.4	2553.7	1715.7
chrX:8440844-8443508	Xp22.31, intergenic	1639	12112	151.2	59.2	30.0	45.5
**chrX:8505855-8509075**	*KAL1*:IVS9-IVS10	4319	14875	147.9	106.6	14.8	44.4
**chrX:9613498-9617784**	*TBL1X*:IVS4	4522	20294	429.2	164.6	115.6	133.7
**chrX:9685383-9689409**	*TBL1X*:Ex18	3938	43044	336.8	101.4	65.5	90.3

Probes from 93 genomic regions exhibiting DA (bold) or equivalent accessibility by metaphase FISH listed by chromosome number and GRCh37 genomic coordinates. Single copy intervals marked with * were characterized by FISH in this study; the other intervals were previously reported.^20–25^ Single copy probes that overlapped genes are specified relative to their exonic (Ex), intronic (IVS) or untranslated (UTR) positions. Single copy probes from intergenic regions were specified by cytogenetic location. Integrated signal intensities of the open chromatin features from ENCODE ^27^ are shown. As appropriate, values are shown with one significant digit after the decimal.

A potential alternative explanation is that differences in probe fluorescence might be related to polymorphic copy number differences in the genome. The genomic intervals covering each of the probes were examined for common copy number variants (CCNV) in the normal population. Two probes within the same genomic interval (*CDK11B*:IVS6; Table 1) overlapped a ~55 kb CCNV (chr1:1,616,989-1,672,591[GRCh37]), but neither exhibited DA. The remaining single copy probes (Table 1) either did not overlap any CCNVs or were known to overlap pathogenic CNV intervals. Population CCNVs cannot account for hybridization intensity differences between homologous chromosomes.

### Chromatin accessibility to homologous metaphase chromosomes is non-random for most differentially accessible targets

FISH probes from chromosomes 1, 5, 9, 11, 15, 17 and 22 showing DA were hybridized to patient samples, in which specific homologs could be distinguished by the presence of a chromosome rearrangement (e.g. a translocation, inversion or heteromorphism) (Table 2). We investigated whether the same homolog in a sample was more likely to have a brighter probe hybridization signal than its counterpart (e.g. non-random), or whether hybridization intensity differences were random (e.g. the brighter signal occurred with equal frequency between homologs).

**Table 2:** **Cell lines and single copy FISH probes used to assess chromatin accessibility**

**Sample ID: cytogenetics**	**Probes for tracking homologs**	
	**Cytoband, gene: interval**	**Status**
GM10958: 46,XX, t(1;11) (q31.2;q25) pat	1q43.3, *RGS7*:IVS4-IVS5 1p36.3, intergenic	DA Equivalent
GM10273: 46,XX, t(11;22)(p13;q12.2) pat	22q13.3, *ACR*:Ex1-IVS3	DA
GM01921: 47,XY, t(8;14)(q13;q13), inv(9)(p11q13) mat, +21	9q34.3, *CACNA1B*:Ex29-IVS31 9p24.3, *C9orf66*:Ex1	DA Equivalent
GM06326: 46, X, t(Y;17) (q11.21;q21) pat	17p12, *PMP22*:IVS3 & *ADORA2B*:IVS1 17p12, *PMP22*:IVS4-Ex5 & *ADORA2B*:Promoter-Ex1	DA Equivalent
GM10958: 46,XX, t(1;11) (q31.2;q25) pat	11q25, *OPCML*:IVS1	DA
GM10273: 46,XX, t(11;22) (p13;q12.2) pat	11q25, *OPCML*:IVS1	DA
II-2: 46,XX.ish del (15) (q11.2q13) (D15S10-,*UBE3A*-) pat	15q12, *SNRPN:*Promoter:IVS1 & *GABRA5*:IVS3 15q13.1, *HERC2*:IVS12-IVS13	DA DA
III-1: 46,XY.ish del(15) (q11.2q13) (D15S10-,*UBE3A*-) mat	Same as II-2	DA
III-2: 46,XX.ish del(15) (q11.2q13) (D15S10-,*UBE3A*-) mat	Same as II-2	DA
L12-1980: 46,XX, t(1;17) (p10;q10)	1q43.3, *RGS7*:IVS4-IVS5 17p12, *PMP22*:IVS3 & *ADORA2B*:IVS1	DA DA
L13-72: 46,XX,9qh+	9q34.3, *CACNA1B*:Ex29-IVS31	DA
L11-729: 46,XY, t(7;22) (q32;q13.33)	22q13.3, *ACR*:Ex1-IVS3	DA

Cytogenetic nomenclature for each of the samples is indicated. Parental origins of the rearrangements are indicated when known (mat = maternal, pat = paternal). Cells are from human lymphocytes (L12-1980, L13-72, L11-729) or lymphoblastoid cell lines [GM10958, GM10273, GM01921, GM06326, and family II-1 (mother), III-1 (child), III-2 (child)].

**Figure 2:**
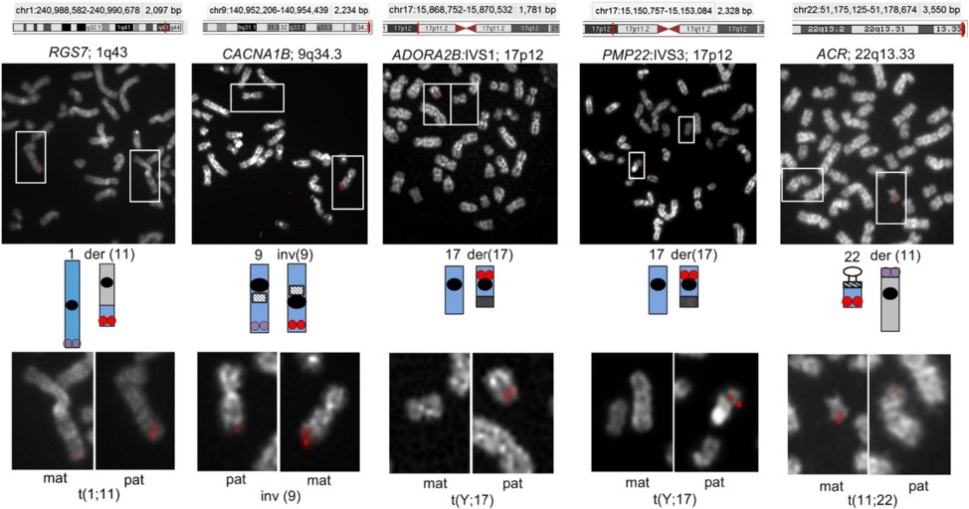
**Detection of DA within cytogenetically-distinguishable homologous regions of known parental origin.** Genomic coordinates of single copy probes detecting DA within 5 different chromosomal regions are indicated. Schematic of the normal and derivative (der) or inverted (inv) chromosome with homologous target are shown. Specific chromosomes are highlighted (white rectangles), ‘mat’ and ‘pat’ refer to the maternal or paternal origin of the altered homolog, respectively. Brighter probe intensity was recurrently observed on the same homolog for a probe for each cell line. *RGS7* probe had greater target accessibility on the der chromosome 11 (paternal, GM10958). *CACNA1B* had greater target accessibility on the inv chromosome 9; (maternal, GM01921). *ADORA2B*:IVS1 and *PMP22*:IVS3 hybridizations were brighter on the derivative chromosome 17 (paternal, GM06326) and *ACR*:Ex1-IVS3 hybridizations were brighter on the normal chromosome 22 (maternal, GM10273).

**Figure 3:**
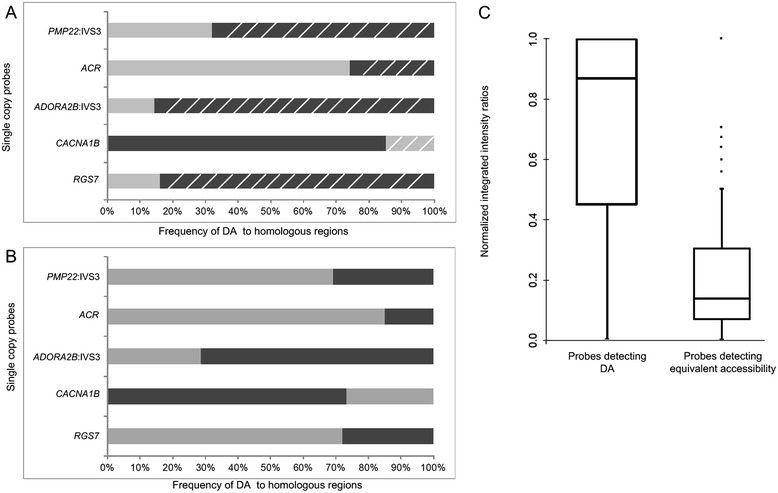
**Differential accessibility is non-random and reproducible between individuals. A**. The light gray and black shading represents the brighter hybridization to either the normal or abnormal homolog, respectively (hatched marks indicate the *paternal* homolog). Bars depicting higher percentages correspond to the more accessible, brighter homolog in a given cell. This was the abnormal paternal homolog for *RGS7* (sample ID: GM10958), abnormal maternal for *CACNA1B* (GM01921), abnormal paternal for *ADORA2B*:IVS1, and *PMP22*:IVS3 (GM06326), and normal maternal homolog for *ACR* (GM10273). **B**. Non-random DA was confirmed using cells from individuals in which the parental origin of the specific chromosomal rearrangement was unknown. The light gray and black shading represents the brighter hybridization to either the normal or abnormal homolog, respectively. Bars depicting higher percentages correspond to the more accessible, brighter homolog in a given cell. *RGS7* probe had greater probe target accessibility on the normal chromosome 1 (sample ID: L12-1980). *CACNA1B* had greater accessibility on chromosome 9 with heteromorphic variant (L13-72). *ADORA2B*:IVS1 and *PMP22*:IVS3 probes were brighter on the abnormal and normal chromosome 17s, respectively (L12-1980) while *ACR* showed greater accessibility to the normal chromosome 22 (L11-729). **C**. Quantification of probe signal fluorescence between homologs are shown by box plots of normalized integrated fluorescence intensity ratios. Single copy probes detecting DA (*RGS7*, *CACNA1B*, *PMP22*:IVS3, *ADORA2B*:IVS1, *ACR*) exhibited large differences in hybridization intensities between homologs. This is indicated by the broad inter-quartile range of normalized intensity ratios from 0.55-1 (median intensity ratio, 0.87). By contrast, normalized intensity ratios for single copy FISH probes (*CCNB1*, *Corf66*, *PMP22:*IVS4-Ex 5, *ADORA2B*:Promoter-Ex1 and 1p36.3 intregenic region) with equal accessibility ranged from 0.07-0.31 (median intensity ratio, 0.14). Intensity differences between homologs were quantified by GVF from 125 metaphase cells for each probe category.

Single copy probes from within genomic regions overlapping *RGS7*, *CACNA1B*, *PMP22:*IVS3, *ADORA2B:*IVS1, and *ACR* showed preferential hybridization (based on probe fluorescence intensity) to the same homologous chromosome in different cells (non-random, p <5.0E-02, two proportion z-test; average of 80% metaphase cells [range 68-86%], n = 30–50 cells, Figures 2 and 3A). Interestingly, non-random DA was noted within *PMP22*:IVS3 and *ADORA2B*:IVS1, while adjacent single copy probes targeting different portions of these same genes (*ADORA2B*:Promoter-Ex1, *PMP22*:IVS4-Ex5) showed similar hybridization intensities (e.g. equivalent accessibility) between homologs. Control single copy probes from within *CCNB1* (Figure 1B, left panel), *C9orf66* (Figure 1B, middle panel), and an intergenic region within 1p36.3 also exhibited equivalent accessibility between homologs. DA is not exclusive to chromosomes originating from one parent-of-origin. For example, single copy probes from within *CACNA1B* and *ACR* exhibited greater accessibility (i.e. brighter fluorescent intensities) to the maternally-derived chromosomal target, whereas *RGS7, ADORA2B:*IVS1, and *PMP22*:IVS3 exhibited increased accessibility to the paternally-derived homolog (Figures 2 and 3A).

The non-random nature of DA was confirmed in a set of independent samples (L12-1980, L13-72, L11-729, Table 2) with distinguishable homologs (Additional file 1: Figure S1), of which parental origins were not known. Non-random DA was observed for probes from within *RGS7*, *CACNA1B*, *PMP22:*IVS3*, ADORA2B:IVS1* and *ACR*, in which the accessible homolog exhibited significantly brighter probe hybridizations (p <5.0E-02; average of 74% metaphase cells [range 69-85%], n =25-50 metaphases per cell line, Figure 3B). Single copy probes from within *PMP22*:IVS3 (in cell line, GM06326) and *RGS7* (GM10958) showed the brighter probe signal hybridized to the abnormal (i.e. derivative) chromosome homolog in the majority of cells analyzed (Figure 3A). By contrast, the same probes when mapped to an additional cell line with a structural alteration (L12-1980), showed that the normal chromosome homolog (Figure 3B) had a more intense hybridization signal. This indicates that DA is not influenced by the presence of particular chromosome rearrangements. Although chromatin accessibility for most DA targets exhibited a non-random preference for one homolog, one DA probe (*OPCML*; 2.53 kb) had a random pattern. This finding was confirmed on two different cell lines with cytogenetically distinguishable chromosome 11s (Table 2 and Additional file 1: Figure S1).

**Figure 4:**
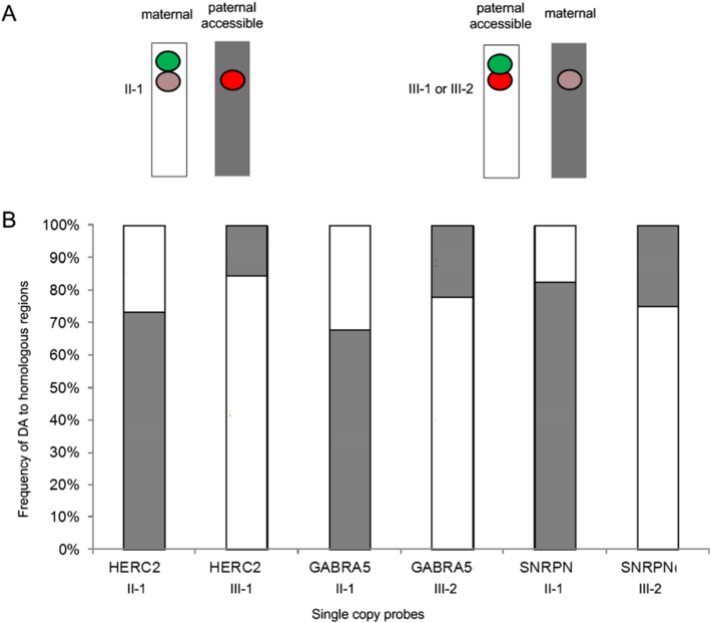
**Differential accessibility is non-random among related individuals. A**. Schematic of a two probe two color single copy FISH strategy to distinguish chromosome 15 homologs is shown. The hemizygous deletion on proximal chromosome 15q is identified by the loss of probe *UBE3A* (green) on one homolog and the presence of *HERC2*, *GABRA5*, *SNPRN* (red, pink). The deletion occurs on the paternal homolog in individual II-1 (mother) and on the maternal homolog in the children (III-1 and III-2). DA for probes outside of the deletion is represented by a bright hybridization on one homolog (red circle) and weak fluorescence hybridization on the other one (pink circle). The deleted chromosome is gray and the normal chromosome is white. **B**. DA detected by *HERC2*, *GABRA5*, *SNPRN* showed that the paternal chromosome in the three individuals (deletion in II-1; normal in III-1 and III-2) contained the brighter fluorescence intensities (*HERC2* II-1, 73.3% of metaphase cells III-1, 84.6%; *GABRA5* II-1, 68% III-2, 77.8%; *SNRPN* II-1, 82.6% III-2, 75.0%) and was more accessible.

We also examined if DA was heritable in 3 members of an Angelman Syndrome (AS) family with a chromosome 15q12 microdeletion (Table 2) at loci adjacent to the rearrangement [13,26]. In this family, the unaffected mother (II-1, Figure 4) inherited the microdeletion from her father (not available for study); and passed on the deleted chromosome to her AS children (III-1, III-2, Figure 4). A dual probe-dual labeling and color detection FISH strategy (Figure 4A) was utilized to distinguish the chromosome 15 homologs based on the presence or absence of the microdeletion. A 4.9 kb single copy FISH probe within the deletion interval (*UBE3A*:IVS7-IVS8, Table 2) served as a control (green circle in Figure 4A) to track the abnormal chromosome 15. Single copy probes detecting DA (dark and light red circles in Figure 4A) targeted intact sequences outside the deletion interval that occurred both within the AS imprinted domain (*GABRA5* [2.77 kb], *SNRPN* [2.09 kb]) and adjacent to the imprinted domain (*HERC2* [1.81 kb]). Irrespective of their imprinted status, probes within *GABRA5*, *SNRPN*, and *HERC2* all showed a bias in non-random hybridization. The paternally inherited chromosome 15, which was deleted in II-1 and intact in III-1 and III-2, consistently exhibited greater probe accessibility (Figure 4B). Previously, we have reported biased early-replication during S phase at the same loci on the paternally-derived chromosome [13]. The variance in the fraction of cells reported to have DA among different samples (Table 2) for all single copy probes described above (*RGS7*, *CACNA1B*, *OPCML*, *GABRA5*, *SNRPN*, *HERC2, ADORA2B*:IVS1, *PMP22*:IVS3, and *ACR*) was not significant (σ^2^ = 9.72, p = 8.65E-01, μ = 35 cells analyzed per sample, Bartlett’s test for homogeneity of variance).

### Quantification of hybridizations confirm variation in fluorescence intensities between homologs for probes detecting DA versus equivalent accessibility

The extent of variation in DNA probe hybridization intensity between homologs was quantified by gradient vector flow (GVF) analysis for both DA probes *(RGS7*, *CACNA1B*, *PMP22*:IVS3, *ADORA2B*:IVS1, *ACR*), and control probes with equivalent accessibility (*CCNB1*, *C9orf66*, *ADORA2B*:Promoter-Ex1, *PMP22*:IVS4-Ex 5, and 1p36.3 intergenic region). Significant differences in integrated fluorescence intensities between homologs with DA were found relative to probes detecting equivalent hybridization (p <5.0E-02; n = 250 total metaphases, Figure 3C). The normalized intensity ratios between homologs in metaphase cells with DA were more variable (σ^2^ = 0.111, μ = 0.716) than control probes with equivalent accessibility to homologous targets (σ^2^ = 0.049, μ = 0.221).

### DA is related to differences in internal chromatin accessibility of homologous targets

**Figure 5:**
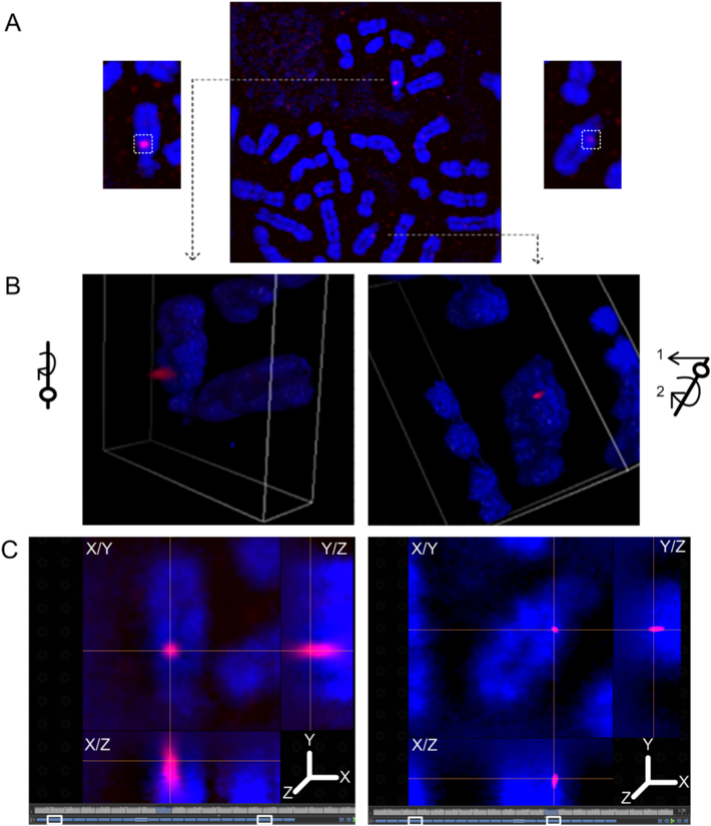
**Visualization of metaphase chromosome differential accessibility in 2- and 3-dimensions. A**. Epifluorescence image of metaphase cell hybridized with *HERC2* single copy probe (1.81 kb) shows a DA pattern. Chromosome 15 homologs are magnified. 3D structured illumination microscopy of hybridized probe volume **(panel B)** and probe depth **(panel C)** for the magnified homologs in **panel A** are presented. **B**. The left homolog with greater accessibility contains fluorescence embedded within the chromosome and protrudes above the surface. In contrast, the right homolog with less accessibility has a much smaller volume of hybridized probe fluorescence and is mainly embedded within the chromosome. Reconstructed volume view in the left homolog was generated by rotating it clockwise about the z-axis (see orientation schematic). Volume view in the right homolog was generated by up-righting it (arrow 1) and turning it clockwise (arrow 2) (see schematic). **C**. Crosshairs are centered over the maximal fluorescent intensity projection along the XY, XZ and YZ axes for each chromosome 15 homolog, and highlight differences in chromatin accessibility. The axial projection (depth) of the probe fluorescence spans 18 of 21 0.1 μm reconstructed optical sections (white rectangles delineate boundaries along the z axis) in the left more accessible homolog; and only 12 of 21 reconstructed optical sections in the right homolog (white rectangles).

**Figure 6:**
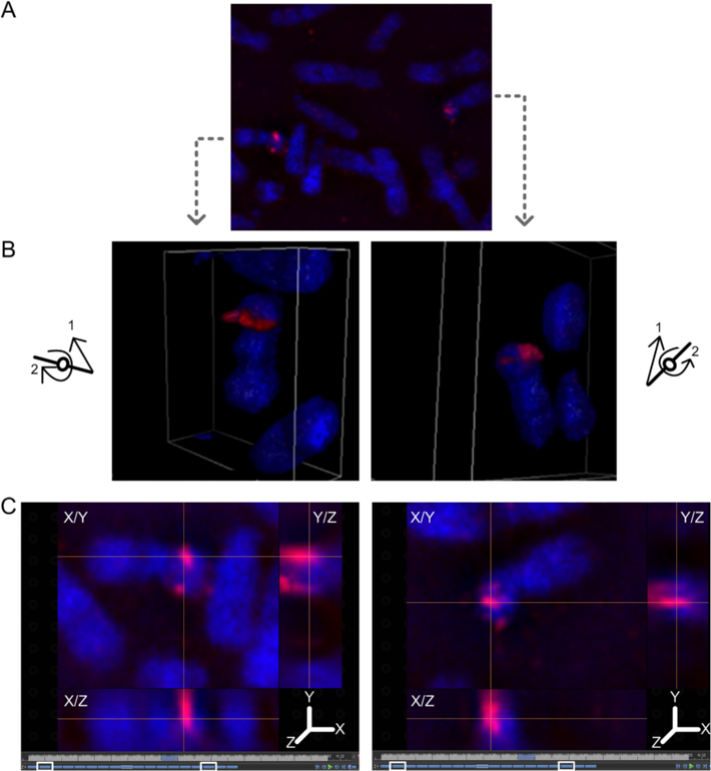
**Visualization of metaphase chromosome equivalent accessibility in 2- and 3-dimensions. A**. Epifluorescence image of metaphase cell hybridized with a low copy probe (3.4 kb) within *NOMO1*. 3D structured illumination microscopy of hybridized probe volume **(panel B)** and probe depth **(panel C)** for the homologs in **panel A** are presented. **B**. Both homologs show equivalent hybridization accessibility, where the fluorescence is embedded within the chromosome and protrudes above the surface. Reconstructed volume view in the left homolog was generated by up-righting it (arrow 1) and turning it clockwise about the z-axis (arrow 2) (see orientation schematic). Volume view in the right homolog was generated by up-righting it (arrow 1) and turning it counter-clockwise (arrow 2) (see schematic). **C**. Crosshairs are centered over the maximal fluorescent intensity projection along the XY, XZ and YZ axes for each chromosome 16 homolog. The axial projection (depth) of the probe fluorescence spans 15 of 18 0.1 μm reconstructed optical sections for both homologs, depicting equivalent chromatin accessibility (white rectangles delineate boundaries along the Z axis).

Using super-resolution, 3-dimensional structured illumination microscopy (3D-SIM), we demonstrated reproducible and significant differences in probe volume (p = 3.72E-07, n = 22 metaphase cells**)** and depth (p = 1.41E-07, n = 22) between homologous regions of three DA probes (*PMP22*:IVS3*, HERC2, ACR*). The distribution of probe volume and depth was broad in regions with DA (Additional file 2: Figure S2A) relative to those with equivalent accessibility (Additional file 2: Figure S2B). For example, a 1.81 kb single copy probe detecting DA within *HERC2* (Figure 5A) exhibited a large difference between homologs (Figure 5B, 0.22 μm^3^ left panel and 0.001 μm^3^ right panel). Notably, the axial distributions (i.e. depth) of the probe fluorescence from the accessible (Figure 5C, left panel) and less accessible (Figure 5C, right panel) homologs were 1.70 μm and 0.80 μm, respectively. These differences in volume and depth projections can also be viewed by traversing through cross-sections of the hybridized chromosomes (Additional file 3: Movie S1, probe *PMP22*:IVS3). The hybridization signals of accessible and DA probes were contained within different focal planes of metaphase chromatin, and there was large variation in the number of reconstructed optical sections hybridized to the same target on different homologs (Figure 5C). By contrast, a probe detecting 5 distinct targets on chromosome 16 (*NOMO1*, Figure 6A) with equivalent accessibility to both homologs showed similar probe volumes (Figure 6B, 0.60 μm^3^, left panel and 0.89 μm^3^, right panel) and depths (Figure 6C, 1.4 μm both panels) (also see Additional file 4: Movie S2). Hybridization to each of these low copy targets were assessed for volume and depth differences as a single fluorescent target due to their close genomic proximity (~1 Mb apart). Among all cells, differences in *NOMO1* probe volume (p = 1.30E-01, n = 20 metaphase cells analyzed) and depth (p = 8.90E-01, n = 20 metaphase cells) between homologs were not significant (Additional file 2: Figure S2B). These findings provide direct evidence that DA is due to the genomic target sequence being less accessible on one of the chromosome homologs.

### Epigenetic features of open chromatin are enriched in genomic regions exhibiting equivalent accessibility versus those with DA

**Figure 7:**
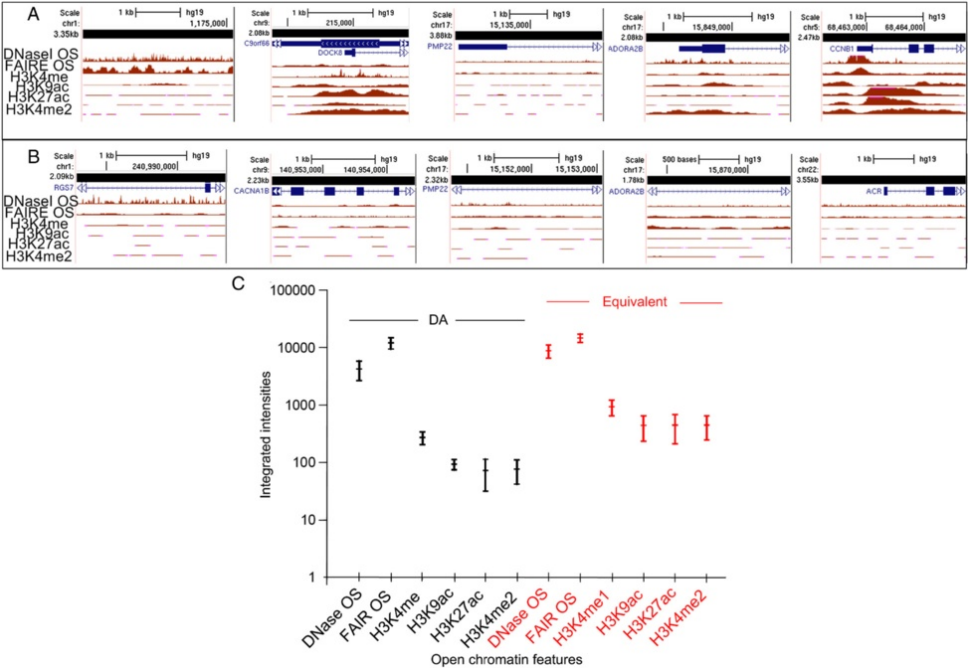
**Correspondence of metaphase chromosome accessibility with epigenetic marks associated with open chromatin in interphase.** Genome browser tracks show integrated ChIP-seq signal intensities of open chromatin features (y-axis) determined by ENCODE. Genomic locations for a set of representative single copy probe intervals is provided (GRCh37) along x-axis, probe size in kilobase pairs is represented by black bar, and genes are shown in blue. **A**. Genomic regions with equivalent accessibility show a higher density of open chromatin epigenetic features than regions with DA **(panel B)**. **C**. The distributions of integrated intensities for each open chromatin feature were plotted around the 95% confidence interval for all probe intervals provided in Table 1, and grouped according to whether the probes showed DA (black bars) or equivalent accessibility (red bars). Group means of the integrated intensity values are shown on the y-axis (y = log 10) and individual features of open chromatin are indicated on the x-axis. The mean integrated ChIP-seq intensities of open chromatin features were significantly different by ANOVA (p =1.0E-04), in particular for all histone marks and DNase I HS, between DA and sequences with equivalent accessibility.

The source of the differences in single copy FISH probe accessibility between metaphase homologs is not known, however other markers of localized, sequence specific chromosome accessibility during interphase are well established [27]. We compared common epigenetic chromosomal modifications diagnostic for open chromatin during interphase to the same genomic intervals that show DA or equivalent accessibility in metaphase (n = 93 genomic regions, Table 1). Interphase epigenetic patterns for single copy intervals detecting equivalent probe accessibility to both homologs showed higher integrated signal intensities. In particular, Deoxyribonuclease I hypersensitivity (DNase I HS), and open chromatin features marked by modifications such as Histone 3 lysine 4 mono-methylation (H3K4me1) and Histone 3 lysine 27 acetylation (H3K27ac) (Figure 7A). These targets exhibited higher integrated signal intensities for DNase HS and histone marks of open chromatin than other marks associated with transcriptionally active chromatin (i.e. H3K36me3, H4K20me1). By contrast, homologous chromosomal intervals exhibiting DA generally had lower integrated signal intensities for the same open chromatin features (Figure 7B), which would be consistent with diminished levels of open chromatin marks at less accessible metaphase loci. Collectively, the average integrated signal intensities of all open chromatin marks (DNase I HS, FAIRE, H3K4me1, H3K9ac, H3K27ac, H3K4me2) in the DA genomic intervals was significantly lower (μ = 2830, σ = 1900) relative to intervals with equivalent accessibility (μ = 4330, σ = 3650) (F = 62.28, p = 1.0E-04; Figure 7C and Table 1).

## Discussion

We have demonstrated differences in accessibility of allelic genomic targets in homologous metaphase chromosomes using independent and complementary approaches. First, we have detected and characterized DA with short, single copy FISH probes in genomic regions representative of telomeric, pericentromeric and chromosome arms (*RGS7*, *CACNA1B*, *PMP22*:IVS3, *ADORA2B*:IVS1, *ACR*, *HERC2*, *GABRA5*, and *SNRPN*) on cytogenetically distinguishable homologs. Differences in probe accessibility between homologs were non-random, and these findings were unrelated to the presence of chromosomal rearrangements that were used as markers to distinguish the homologs. With the one exception (*OPCML*), the brighter signal for each of the probes exhibiting non-random DA was biased to the same homolog in the cells from an individual. At the *OPCML* locus, DA occurred randomly, with either homolog exhibiting greater accessibility.

Aside from non-random hybridization patterns, DA was also found to be heritable. The proximal 15q region showed greater accessibility on the paternally-derived homolog, irrespective of the presence of a small molecular deletion adjacent to these probes. This pattern was stable and preserved across two generations in a family carrying the deletion. While our results do not inform on the degree to which parent-of-origin effects contribute to DA, future studies of additional familial rearrangements of known parental origin (e.g. chromosome 11;22 translocation carriers) for the probes in this study, as well as others, will be useful in demonstrating this.

The three dimensional distribution of probes displaying DA was visualized by 3D-SIM. This technique improves optical resolution by two-fold over conventional imaging, and more precisely delineates probe signals. Imaging at sub-optical diffraction scale occurs at a much higher frame rate, which enabled us to quantify differences in chromatin structure between homologous regions for single copy FISH probes more efficiently relative to other super-resolution techniques [5,28,29]. The spatial distributions of fluorescent hybridization to chromosome targets, emitted by single copy probes with DA, varied between homologous metaphase regions. The homolog with a lower hybridization intensity signal exhibited restricted probe occupancy in both the lateral and axial dimensions. The depth of the target sequences on the less accessible chromosome was also found to be an order of magnitude less than its corresponding homolog in the same cell. Finally, the target sequence in the homolog with lower intensity hybridization occupied a smaller volume of metaphase chromatin based on the spatial distribution of its probe fluorescence. The radial chromosome structure hypothesis, suggests that accessibility should be related to the proximity of the target sequence to the chromosome surface [30]. Our results suggest rather, that the differences in the volume and depth of the hybridized target sequence are more likely related to the degree of compaction of corresponding DNA in each of the homologous chromosomes.

Based on our ENCODE analysis of genomic regions with DA or equivalent accessibility (Figure 7 and Table 1), we envision that the differential condensation of homologous chromosomes represents a transition between parental and daughter cell epigenetic states. Histone marks and chromatin binding proteins may potentiate some genomic loci to maintain a less condensed configuration of one or both alleles during metaphase, which might then poise them to restructure open chromatin regions during the subsequent interphase in daughter cells [31–36]. This transition state may be akin to a type of chromatin memory that recalls epigenetic marks derived from the preceding interphase so that they can be transmitted and re-established in subsequent daughter cells. To assess DA as a means of storing chromatin memory will be technically challenging. Chromatin modifications catalyze dynamic structural changes that arise over the course of interphase. It would be necessary to score DA at different cell cycle stages (e.g. G1, S, G2) to place these results in context. This would require enriched, synchronized cell populations at the end of G2 still possessing markers of interphase chromatin at the inception of chromosome condensation. Only a small fraction of unsynchronized cells are in G2. Interphase analysis was beyond the scope of the present study which was to demonstrate and characterize DA on mitotic metaphase chromosomes.

Reduced DNA accessibility may affect chromatin structure and histone modification (the most extreme instance being X chromosome inactivation), enabling the cell to maintain control over epigenetic variation in regulatory regions [37,38]. This mechanism could exclude co-regulation of both allelic regions at a DA locus [39]. Differences in chromatin accessibility may be a way to distinguish and spatially organize homologous loci so that the less accessible locus is separated from its accessible counterpart. To this end, homologous chromosomes are known to be in repulsion, e.g. significantly more distant from one another in the interphase nucleus relative to heterologous pairs [40]. Alternatively, DA could be envisioned as a stepwise process of chromosome condensation that packages DNA into highly condensed polymers in a tightly confined space [41], producing heterogeneous levels of compaction, as we have observed at discrete allelic loci.

Specific epigenetic marks such as histone modifications or topological constraints on chromatin that characterize each allele at the same locus may be a mechanism that underlies DA. Epigenetic marks can be propagated to ensure stability of chromatin memory and cellular identity in daughter cells, following mitosis [42]. Our findings can be interpreted in this context. Previous studies have demonstrated retention of nuclease hypersensitivity, transcription factor occupancy, and selective histone marks on mitotic chromatin [31–36]. Tri-methylation of histone H3 on lysine 9 and 27 is stably transmitted through interphase including mature post-replicative chromatin [43]. Differential condensation of homologous chromosomal regions could encode these features in a structural form that effectively memorizes the state of chromatin preceding metaphase. Maintenance of chromatin memory would be important for normal development and disease avoidance [43].

Previous work has demonstrated differences in intrachromosomal compaction using large FISH probes (e.g. cosmids or bacterial artificial chromosome [BAC] based probes) hybridized to a complex mix of chromatin fibers [44]. Reproducible differential hybridization patterns between metaphase homologs over short genomic distances (Table 1) have not been previously reported. The probes used to demonstrate DA are distinct from short single copy oligonucleotide (25–50 basepairs) DNA probes [45], densely tiled along a particular genomic region of ≥25 kb in length, that produce fluorescence signal intensities equivalent to a cosmid or a BAC. The differences in hybridization intensities to homologous chromosome regions of tiled oligonucleotides or large recombinant DNA probes are much less pronounced than the contiguous single copy probes used in the present study. BAC-based FISH probes, therefore, are not as sensitive for detection of DA, as these probes likely contain both genomic intervals with equivalent accessible and DA targets, and their longer target length increases their overall fluorescence intensity.

We have combined single or low copy probes for FISH, which together are on average 10 kb or more in genomic length, to assess boundaries of chromosomal rearrangements in complex genomic architecture [20,21,24]. The total length of these genomic targets does not solely dictate signal intensity. Probes of similar length and composition can vary in fluorescence intensity when hybridized to different regions in the human genome [20,21]. In the present study, a 3.5 kb probe detects DA on chromosome 22 within *ACR* (Figure 2)*,* whereas a smaller 2.08 kb single copy probe within *C9orf66* (Figure 1B) shows equivalent accessibility and bright signals to both homologs. In addition, a low copy probe with 3 distinct genomic targets spanning 8.5 kb within *HERC2* segmental duplicons exhibits DA (Additional file 1: Figure S1F). Finally, we did not find any remarkable differences in the GC content of individual single copy probes exhibiting DA relative to those showing equivalent accessibility (Additional file 5: Table S1). Our findings instead suggest that the context of the chromosomal regions themselves and their respective degrees of condensation primarily determine the differences in hybridization signal intensities that we observe.

## Conclusions

We have previously designed and tested [20,21] novel single copy DNA probes to precisely ascertain small pathogenic chromosome copy number changes and complex genomic architecture in the human genome [24]. In this study, we have expanded the utility of single copy DNA sequences to investigate chromatin accessibility differences between metaphase chromosome homologs. We demonstrate that chromatin accessibility differences are non-random with respect to specific homologous loci, they occur within exons, introns and intergenic regions, and these regions are not enriched for epigenetic marks of accessible interphase chromatin. Examination of allelic regions with DA, by super-resolution 3D-SIM, further showed that the internal chromatin structure of the accessible locus is less condensed relative to its inaccessible counterpart. Expanding the analysis of DA on a genomic scale to larger chromosomal domains containing allelic regions can help generate a high resolution map of chromatin accessibility during metaphase. Relating this information to epigenetic modifications during interphase may provide possible insight into how higher order chromatin structure is remodeled during mitosis.

## Methods

### Probe selection and scoring of differential accessibility (DA) on hybridized metaphase chromosomes

Single copy genome-coordinate defined DNA probes were previously developed and used with FISH to precisely localize breakpoints in rearranged metaphase chromosomes for many different diseases and disorders [20–25]. All single copy probes are devoid of repetitive elements and their nucleotide composition and genomic coordinates are precisely known. They map to a single location and can be developed from any unique region in the genome (e.g. exons, introns, intergenic, regulatory). As part of the development and validation of these single copy probes for FISH, they were hybridized to normal human chromosomes from the lymphocytes of at least one male and one female to confirm mapping of the probes to the expected genomic location [20–25]. Genomic locations of single copy probes were also compared to locations of common CNVs (≥1% of general population) from blood derived DNA in two independent sample sets from healthy individuals. Common CNVs on both sample sets were identified on Affymetrix CytoScan HD array using ChAS (Chromosome Analysis Suite) software. These population CNV data were obtained from Ontario Population Genomics Platform (873 individuals of European ancestry with minimum of 25 probes per CNV; Database of Genomic Variants) and Healthy sample track (~400 individuals with minimum of 35 probes per CNV; obtained from Affymetrix). During our validation studies, it was observed that while most single probes hybridized with similar affinity to both homologs within a cell, there were some probes in the validation samples with consistent, striking probe hybridization fluorescence intensity differences (or differential accessibility [DA]) between homologs. These probes were not pursued for clinical applications. In this study, we revisited some of these probes to begin to characterize the disparate fluorescence intensity differences between homologs. In order to determine if the hybridization intensity patterns were non-random, we selected DA probes based on availability of patient samples with cytogenetically distinguishable homologs (one normal, one rearranged) and the specific chromosomes involved in the rearrangements. Table 2 lists the FISH probes, their chromosomal location and the karyotypic findings of the 10 cell lines used to assess chromatin accessibility. These DA FISH probes were euploid and did not overlap the rearranged chromosomal regions. Parental origin of the chromosome rearrangement was known for 4 cell lines. Three cell lines (II-1 [mother], III-1 and III-2 [children]) were from a family carrying a microdeletion within the chromosome 15q12 imprinted region [13,26]. The remaining cells lines were from unrelated individuals.

### Chromosome preparations and fluorescence In situ hybridization

Peripheral blood lymphocytes or lymphoblastoid cell lines were cultured and chromosomes harvested using routine cytogenetic methods that included 0.075 M KCl hypotonic solution and 3:1 methanol:acetic acid fixation (Carnoy’s fixative) (also see Additional file 6: Supplementary methods) [46]. With the exception of single copy FISH probe designed from within *CCNB1* (2.47 kb) on chromosome 5q13.2 (genomic coordinates, Table 2), all probes were previously developed [20,25]. The *CCNB1* probe was specifically designed from a genomic region with hallmarks of open chromatin [31–36]. Single copy FISH probes used in this study ranged from 1.78 kb to 3.55 kb in length. Details of probe amplification, purification, labeling, hybridization, and detection are provided in supplementary material and have been previously described [47]. To identify the chromosome 15q12 submicroscopic deletion (II2, III-1 and III-2), different biotin-labeled and digoxigenin-labeled single copy probes (one probe from within the deletion and one adjacent to the deletion), were hybridized simultaneously and detected in different colors to distinguish the deleted homolog from the normal one. For the other cell lines, the normal and rearranged homologs were distinguishable by DAPI staining and single copy probe hybridizations were performed.

DA was scored as differences in FISH probe hybridization intensities between homologous loci by direct examination using epifluorescence microscopy, and subsequently by quantification of hybridized probe epifluorescence images. At the microscope, hybridized probe fluorescence signals for each homolog were scored as bright, intermediate, dim, or nil. For a cell to be scored as DA, one homolog was required to exhibit an intermediate or bright probe signal and the other homolog a different intensity signal (e.g. bright/intermediate, bright/dim, bright/nil, intermediate/dim or intermediate/nil on homologs in a cell). For a cell to be scored as having equivalent accessibility, both homologs were required to exhibit probe hybridization of similar intensities (e.g. bright/bright, intermediate/intermediate). Microscope slides with metaphase cells were coded, hybridized and scored by 2 certified cytogeneticists. Twenty-five to 50 hybridized cells were scored for each sample. To exclude bias resulting from inefficient hybridizations, cells with dim hybridizations on both homologs or in which one homolog had a dim hybridization and the other had no hybridization were not scored. A two proportion Z-test was used to determine whether the fraction of cells showing DA or equivalent accessibility was statistically significant at α = 5.0E-02. Variance in the frequency of cells reported to have DA among different samples was assessed for significance (α = 5.0E-02) using Bartlett’s test for equality of variances.

For DA probes, a two proportion Z-test was also used to determine whether there was non-random preference for one parental homolog to have brighter probe fluorescence intensity (i.e. more accessible hybridization). From the Z-test score, a *p*-value was obtained to determine whether the proportion of the brighter hybridizations showed a significant bias (α = 5.0E-02) to one homolog. Additionally, probe fluorescence intensities in each cell were quantified by integrated gradient vector flow (GVF) analysis (next section).

### Gradient Vector Flow (GVF) analysis to quantify differences in probe intensity between homologs

We previously developed a GVF-based algorithm that determined probe hybridization boundaries and quantified probe fluorescence [5,48]. The GVF algorithm generated an active binary contour of the gray scale image of the probe fluorescence on each homolog. From the active contour, the integrated intensity values (in pixels) were calculated. The intensity values were normalized for each cell by taking the difference in integrated intensities between homologs, and dividing this difference by the sum of the intensities of both homologs. This converted raw total intensity values into a set of normalized intensity ratios (0 to 1). Values close to 0 confirmed that the probe intensities between the homologs appeared equivalent and ratios close to 1 indicated DA. A bias in hybridization intensities between homologous regions was reported as statistically significant (α = 5.0E-02) using a two-tailed *t*-test.

### Examination of short target hybridized probe features using high resolution 3-D structured illumination super-resolution microscopy

3D-SIM (Nikon Corporation) was used to examine and quantify volume and depth of single and low copy DNA probe fluorescence embedded in metaphase chromatin. Low copy probes recognize multi-target DNA sequences that occur within segmental duplications [24]. 3D-SIM image reconstruction algorithms, for generating high resolution chromosome images, were optimized using a low copy probe from within *NOMO1* hybridized to normal metaphase chromosomes. This probe yielded bright fluorescence signals on both homologs as it hybridized to multiple genomic targets on chromosome 16 duplicons, ([GRCh37] genomic coordinates: 16452359–16455837, 15013674–15017156, 16412325–16415807, 18440574–18444056, and 18484058–18487536).

Chromosome image acquisition was performed on a motorized inverted Ti-E microscope equipped with a CFI Apo TIRF 100X oil (NA 1.49) objective (Nikon USA) and SIM illuminator (Nikon Corporation) in stack 3D-SIM mode. The epifluorescence image was captured using total internal reflection fluorescence mode followed by 3D-SIM on the same cell to gain resolution in the X/Y/Z dimensions. Compatible lasers with wavelengths of 457 nm and 561 nm were used to excite DAPI (chromosome counterstain) and Cy3 (probe fluorescence), respectively. Using moiré superimposed pattern formation [28], high frequency signal components were captured and deduced from the image reconstruction algorithms. Fast Fourier transforms were generated to validate that previously irresolvable high frequency signals from the epifluorescence metaphase image had been properly acquired by 3D-SIM (Additional file 7: Figure S3). The NIS-Elements AR software (version 4.13.00, Nikon Canada Inc.) reconstructed 3D-SIM images of hybridized sequence-defined probes demonstrating DA (*HERC2*, *PMP22*:IVS3, *ACR*) or equivalent accessibility (*NOMO1*) to metaphase chromosome homologs. The lateral fluorescence depth of each probe was calculated from a maximum of 20 reconstructed optical sections. Each section was collected in 0.1 μm steps from a total of 20 metaphase cells for *NOMO1*, 10 cells each for *HERC2* and *PMP22:*IVS3, and 2 cells for *ACR*. A threshold on the gray scale image of the DNA probe signal was performed in NIS-elements software using image segmentation, which converted the gray scale image into a binary image contour. Following probe fluorescence thresholding, the volume of bound probe fluorescence was calculated over all reconstructed optical sections. From these data, differences in probe volume and depth between homologs were quantified (NIS-Elements AR software) and analyzed for significance (α = 5.0E-02, two-tailed *t* test). Movie montages of DNA probe volume and depth were generated as AVI files, using the Movie Maker option (NIS-Elements AR software). Key frames depicting DA between homologs from all angles were added to the movie in order to emphasize the volume view, which built and rotated the metaphase chromosome 360° around the X/Y/Z axis.

### Sequence analysis of epigenetic chromatin marks for single copy probes detecting DA or equivalent accessibility

The genomic sequence of the single copy probes, which displayed DA or equivalent hybridization accessibility (asterisks, Table 1) in metaphase were compared with epigenomic DNA features that characterize open chromatin and active regulatory elements during interphase in multiple cell types [27,49]. The epigenomic features from ENCODE [27] that we examined include DNase1 HS, Formaldehyde-Assisted Isolation of Regulatory Elements (FAIRE), and histone marks (H3K4me1, H3K9ac, H3K27ac, H3K4me2). The cell line used for ENCODE interphase comparisons, (GM12878, Coriell Cell Repository), was of the same B-cell lineage that we used to characterize DA and equivalent chromatin accessibility on metaphase homologs (Table 2). Furthermore, the cells were grown under the same culture conditions (37°C/5% CO_2_ in RPMI-1640 complete medium with 15% fetal bovine serum). ENCODE chromatin immuno-precipitation sequencing (ChIP-seq) data generated high resolution, multidimensional view of chromatin accessibility from the above-mentioned epigenomic DNA features [50]. ChIP-seq signal intensities of each open chromatin feature were visualized along the full length of a given single copy interval using the UCSC (University of California Santa Cruz) genome browser. Individual data points of the ChIP-seq signal intensities overlapping the genomic length of each single copy interval (Table 1) were retrieved from the UCSC table browser using the Duke DNase1 HS, University of North Carolina FAIRE seq, and Broad Institute histone modification custom tracks. The data point intensities were summed for each single copy interval (Table 1) and mean integrated single intensity values with standard deviations at 95% confidence were computed and plotted for all open chromatin features within each category (DA or equivalent accessibility). We then determined whether the differences in these values were significant by the analysis of variance test (ANOVA) for DA probes versus those with equivalent accessibility. Significance was determined from the *p* value of the F ratio following ANOVA.

## Additional files

Additional file 1: Figure S1 Examples of probes with DA by FISH. Arrows indicate the less accessible homolog (i.e. the weaker hybridization signal). **A-E**. Single Copy Probes: Dim or no hybridization is on the derivative chromosome 1 for *RGS7* (cell line L12-1980), the normal chromosome 11 for *OPCML* (cell line GM10958), the normal chromosome 17 for *ADORA2B*:IVS1 (cell lines L12-1980), the derivative chromosome 17 for *PMP22*:IVS3 (cell line L12-1980), and the derivative chromosome 7 for *ACR* (cell line L12-1989), respectively. The other homolog in each panel has brighter hybridization signals. **F**. Low Copy Probe: *HERC2* duplicon probe detects three distinct paralogous targets spanning 8.5 kb on chromosome 15 s from normal cell. Additional file 2: Figure S2 Quantification of differences in DNA probe volume and depth between probe regions for DA and equivalent accessibility following 3D-SIM. **A**. Genomic targets within *HERC2*, *PMP22*:IVS3, and *ACR* had 3.3-fold greater volumetric, normalized integrated probe intensities (μ =0.72 μm^3^, range: 0.15-1.0 μm^3^, n =22 cells) compared to a genomic target with equivalent accessibility within *NOMO1* (**panel B**, μ = 0.22 μm^3^, range: 0–0.34 μm^3^, n = 20 cells). Genomic targets within *HERC2*, *PMP22*:IVS3, and *ACR* (**panel A**) also had broad distributions of probe depth (range: 0.005-1.0 μm) confirming DA versus genomic targets within *NOMO1* (**panel B**) which showed smaller differences in probe depth (range: 0–0.14 μm), confirming equivalent accessibility between homologous regions. Probe volume and depth were not correlated for genomic regions with DA (r =0.163) and equivalent accessibility (r = − 0.281). Following quantification, normalization for probe volume was performed by subtracting the volumes between homologous targets and dividing by the total probe volume for each cell. Similar normalization was done for probe depth. Additional file 3: Movie S1 3D anaglyph view of single copy FISH probe targets with DA (*PMP22*:IVS3) between chromosome homologs. Movie in upper left panel shows differences in probe fluorescence depth, dynamically visualized through 0.1 μm optical cross-sections of the hybridized chromosome 17 homologs. Upper right panel is a 3D projection of the DNA probe fluorescence, from which probe volume was obtained. The lower panel shows the same homologs, as in upper left, with occupancy of probe volume in the context of the reconstructed chromosomes, rotated 360° in the *X/Y/Z* axes and depicting inter-homolog DA from all angles. Reconstructed optical sections were taken over 20 z-stacks, at 0.1 μm per stack with 3D-Structured Illumination Microscopy. Additional file 4: Movie S2 3D anaglyph view of low copy FISH probe targets (*NOMO1)* with equivalent accessibility between homologs. The upper panels show probe volumes in the reconstructed chromosomes, rotated 360° around the X/Y/Z axes. Lower left panel is a 3D projection of the DNA probe fluorescence from which probe volume was obtained. In lower right panel, *NOMO1* probe fluorescence is shown embedded within the accessible invaginations of metaphase chromatin topography. Chromosome topography was generated by tapping mode raster scanning using atomic force microscopy. Topography and fluorescent probe signals were correlated using overlay procedures previously described (see reference [5]). Reconstructed optical sections were taken over 18 z-stacks, at 0.1 μm per stack. Chromosome 16 homologs shown are from a different metaphase cell than Figure 6. Additional file 5: Table S1 DA probe intervals with chromosome location (column 1), genomic coordinates (columns 2 and 3) and fractional GC content (column 4). GC content was calculated for an interval by obtaining the genomic sequence in FASTA format using the Galaxy Metaserver (url: https://usegalaxy.org/) and then inputting the sequence into Galaxy EMBOSS tool (‘geecee’) to calculate GC percentage. Average GC content for the 34 genomic regions with DA was 47.3% with a low standard deviation (μ = 0.473, σ = 0.08). ‘*’ indicates probes hybridized on cells with distinguishable chromosome homologs in this study to examine random vs nonrandom features of chromatin accessibility. Refer to Table 1 for specific genic regions within each interval. Additional file 6
**Supplementary Methods.** Details of chromosome cell culture, single copy DNA probe preparation, and in situ hybridization are provided. Additional file 7: Figure S3 Validation of super-resolution imaging of metaphase chromosomes before and after 3D-Structured Illumination Microscopy. **A**. Fast Fourier transform (FFT) shows the point spread function from a wide field epifluorescence metaphase with a hybridized single copy probe with DA (*HERC2*, 1812 bp). **B**. FFT on the same cell following 3D-SIM. This verified that the point spread function of super-resolution 3D-SIM was an order of magnitude higher than the wavelengths of wide field epifluorescence, as it captured high frequency measurements of fluorescent objects. This was used as a quality control metric to validate resolution of the 3D-SIM data on the Nikon Ti-E SIM illuminating system.

## Abbreviations

ANOVA: Analysis of variance
AS: Angelman syndrome
ChIP-seq: Chromatin immuno-precipitation sequencing
CCNV: Common copy number variant
DA: Differential accessibility
DNase I HS: Deoxyribonuclease I hypersensitivity
ENCODE: Encyclopedia of DNA elements
FAIRE: Formaldehyde-assisted isolation of regulatory elements
FISH: Fluorescence in situ hybridization
GVF: Gradient vector flow
H3K9Ac: Histone 3 lysine 9 acetylation
H3K27ac: Histone 3 lysine 27 acetylation
H3K4me1: Histone 3 lysine 4 mono-methylation
H3K4me2: Histone 3 lysine 4 di-methylation
3D-SIM: 3-dimensional structured illumination microscopy
