# Supplementary figures and images for: Localized, non-random differences in chromatin accessibility between homologous metaphase chromosomes
Source: Mol Cytogenet. 2014 Nov 19;7:70. doi: 10.1186/s13039-014-0070-y (PMC4269072; doi:10.1186/s13039-014-0070-y)

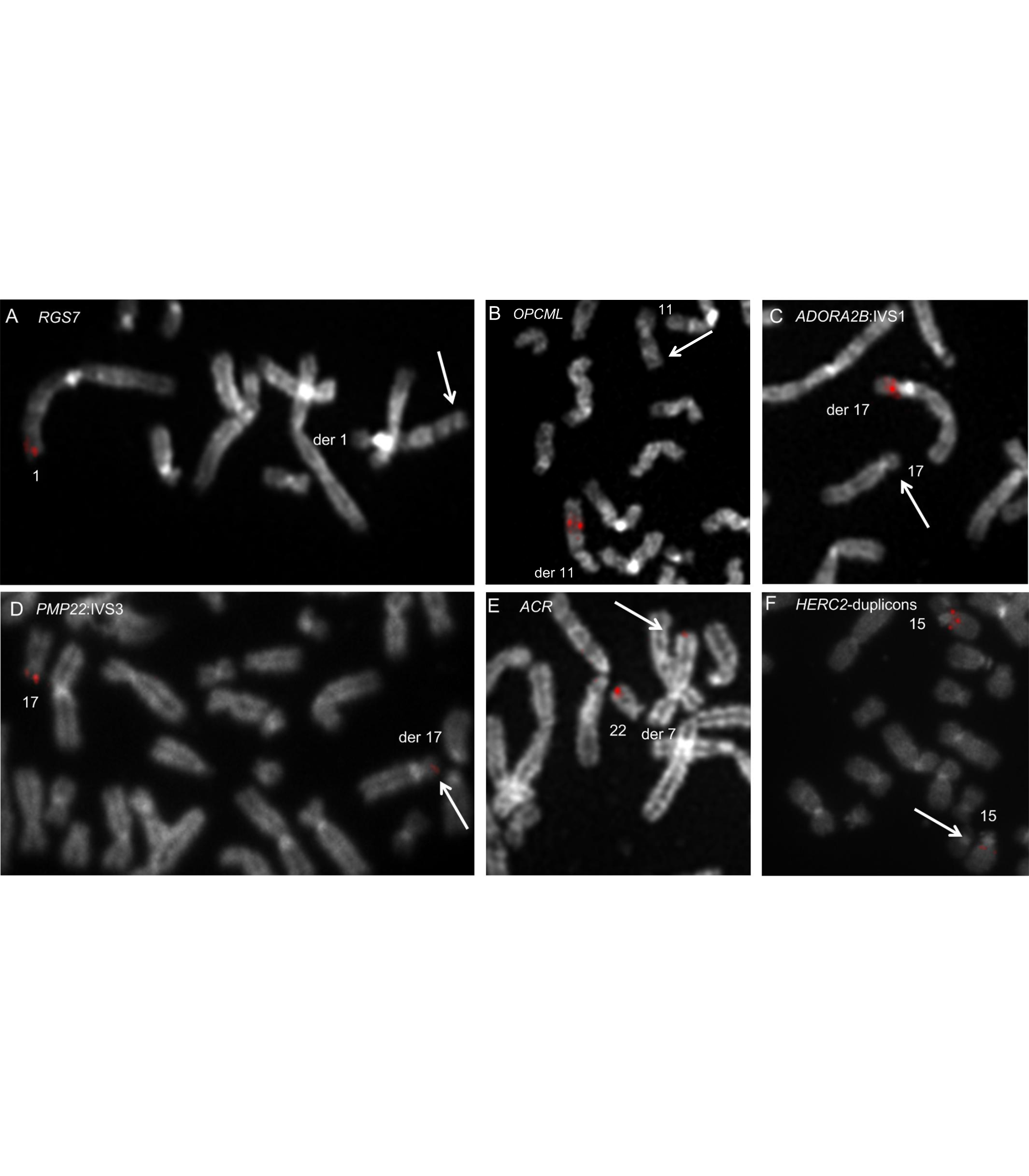

Supplement: Additional file 1: Figure S1 — Examples of probes with DA by FISH. Arrows indicate the less accessible homolog (i.e. the weaker hybridization signal). A-E. Single Copy Probes: Dim or no hybridization is on the derivative chromosome 1 for RGS7 (cell line L12-1980), the normal chromosome 11 for OPCML (cell line GM10958), the normal chromosome 17 for ADORA2B:IVS1 (cell lines L12-1980), the derivative chromosome 17 for PMP22:IVS3 (cell line L12-1980), and the derivative chromosome 7 for ACR (cell line L12-1989), respectively. The other homolog in each panel has brighter hybridization signals. F. Low Copy Probe: HERC2 duplicon probe detects three distinct paralogous targets spanning 8.5 kb on chromosome 15 s from normal cell. [file 13039_2014_70_MOESM1_ESM.tiff]

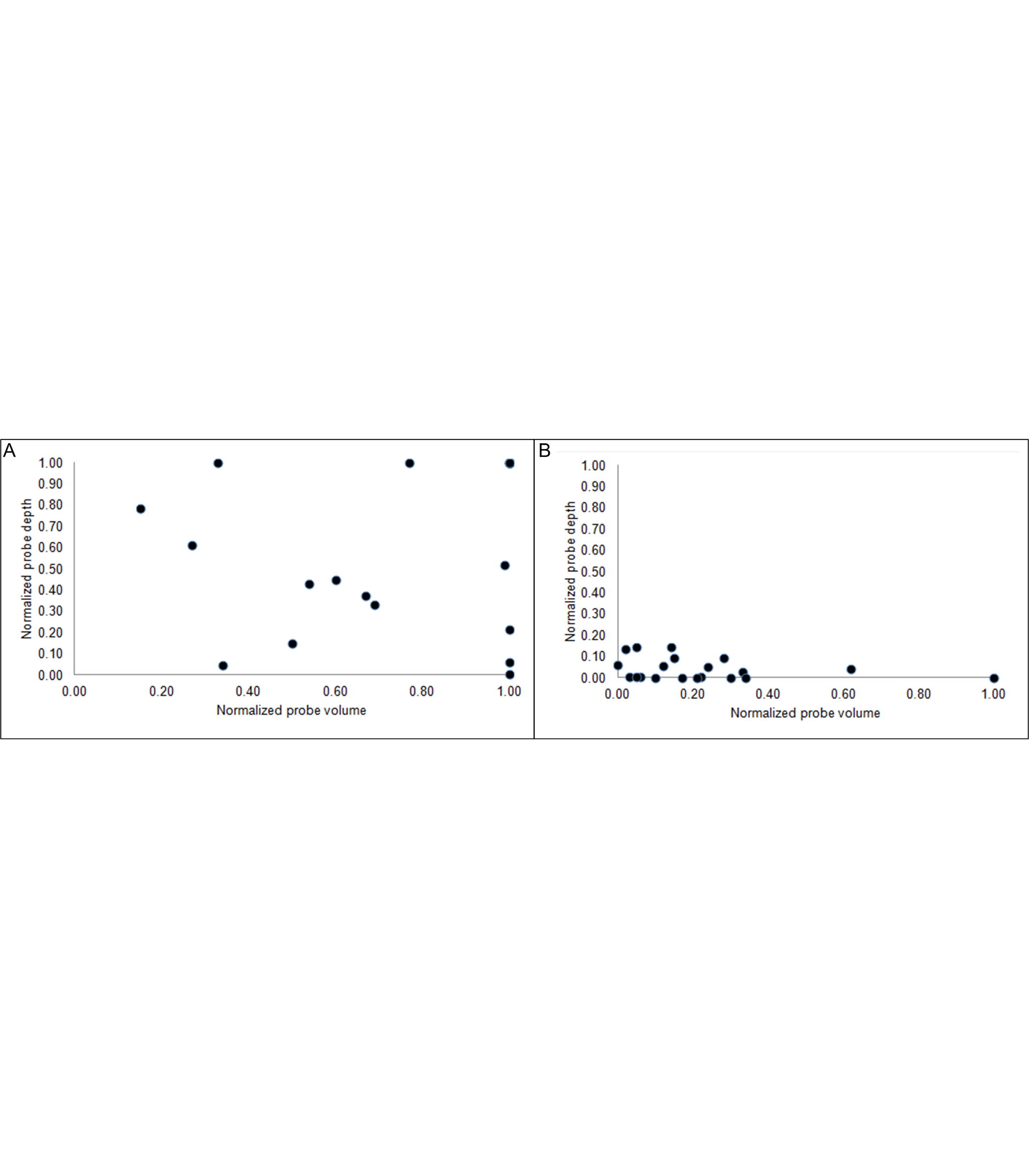

Supplement: Additional file 2: Figure S2 — Quantification of differences in DNA probe volume and depth between probe regions for DA and equivalent accessibility following 3D-SIM. A. Genomic targets within HERC2, PMP22:IVS3, and ACR had 3.3-fold greater volumetric, normalized integrated probe intensities (μ =0.72 μm3, range: 0.15-1.0 μm3, n =22 cells) compared to a genomic target with equivalent accessibility within NOMO1 (panel B, μ = 0.22 μm3, range: 0–0.34 μm3, n = 20 cells). Genomic targets within HERC2, PMP22:IVS3, and ACR (panel A) also had broad distributions of probe depth (range: 0.005-1.0 μm) confirming DA versus genomic targets within NOMO1 (panel B) which showed smaller differences in probe depth (range: 0–0.14 μm), confirming equivalent accessibility between homologous regions. Probe volume and depth were not correlated for genomic regions with DA (r =0.163) and equivalent accessibility (r = − 0.281). Following quantification, normalization for probe volume was performed by subtracting the volumes between homologous targets and dividing by the total probe volume for each cell. Similar normalization was done for probe depth. [file 13039_2014_70_MOESM2_ESM.tiff]

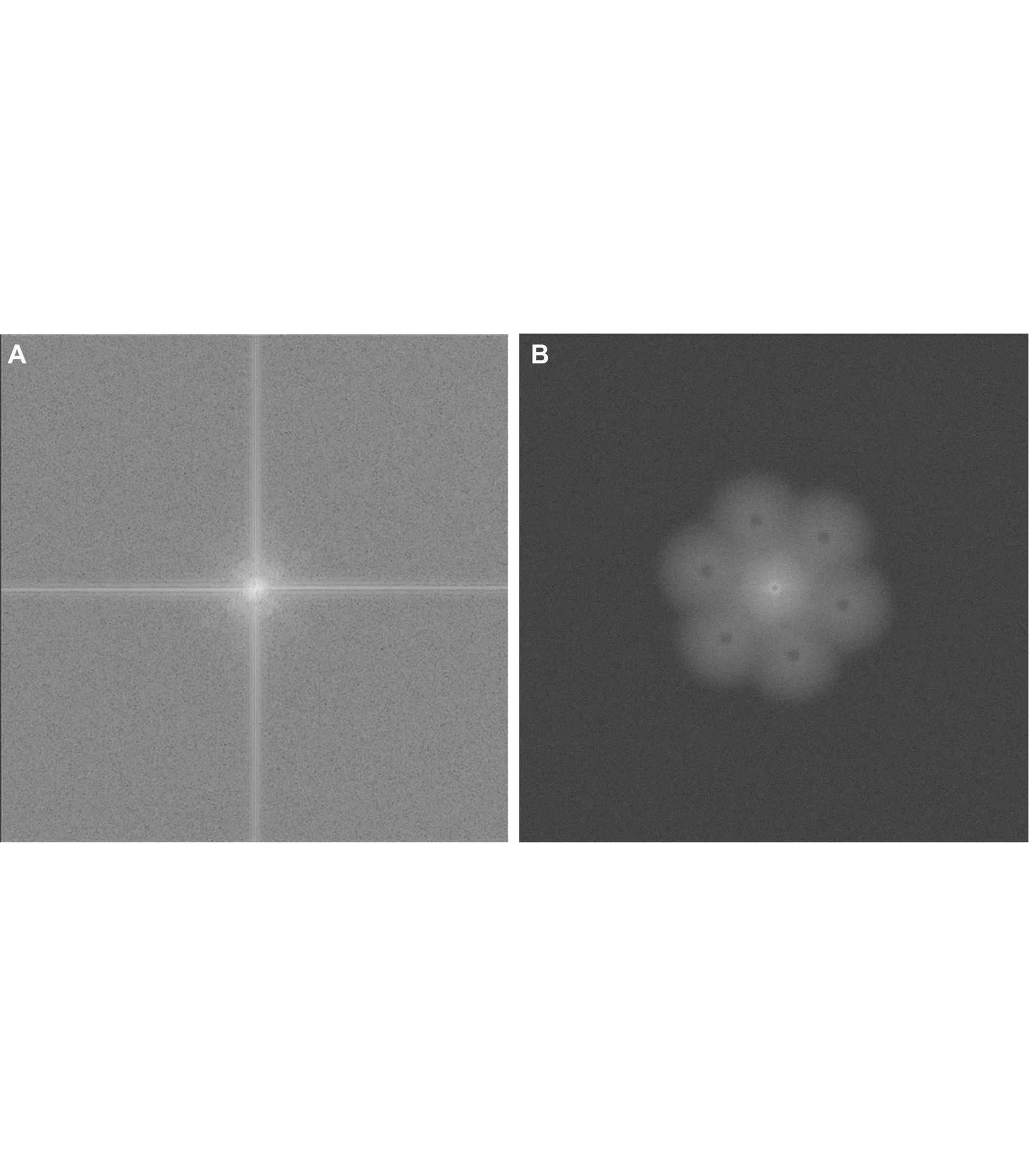

Supplement: Additional file 7: Figure S3 — Validation of super-resolution imaging of metaphase chromosomes before and after 3D-Structured Illumination Microscopy. A. Fast Fourier transform (FFT) shows the point spread function from a wide field epifluorescence metaphase with a hybridized single copy probe with DA (HERC2, 1812 bp). B. FFT on the same cell following 3D-SIM. This verified that the point spread function of super-resolution 3D-SIM was an order of magnitude higher than the wavelengths of wide field epifluorescence, as it captured high frequency measurements of fluorescent objects. This was used as a quality control metric to validate resolution of the 3D-SIM data on the Nikon Ti-E SIM illuminating system. [file 13039_2014_70_MOESM7_ESM.tiff]
