# Supplementary material for: Localized, non-random differences in chromatin accessibility between homologous metaphase chromosomes
Source: Mol Cytogenet. 2014 Nov 19;7:70. doi: 10.1186/s13039-014-0070-y (PMC4269072; doi:10.1186/s13039-014-0070-y)
Supplement: Additional file 6 — Supplementary Methods. Details of chromosome cell culture, single copy DNA probe preparation, and in situ hybridization are provided. [file 13039_2014_70_MOESM6_ESM.docx]

**Supplementary Methods**

***Chromosome preparation***: Lymphoblastoid/lymphocyte cells were cultured in T25 tissue culture flasks in RPMI-1640 medium (Gibco, Life Technologies Inc. ON, Canada) supplemented with L-glutamine, 15% fetal bovine serum (Gibco) and1% penicillin/streptomycin (Gibco). Cells were grown at 37^o^C/5% CO_2_ in a humidified incubator; and harvested in logarithmic growth phase at a concentration of 1x10^6^ cells/ml by arresting cells in metaphase [30μl of 10μg/ml Colcemid, Gibco) in a final volume of 10ml culture medium for 30 minutes. Subsequently, cells were pelleted by centrifugation, resuspended in hypotonic solution (0.075M KCl) for 20 minutes and fixed with 3 parts methanol (ACP Chemicals Inc. ON, Canada) to 1 part acetic acid (Caledon laboratory chemicals, ON, Canada).

***Single copy DNA probe preparation***: All genomic intervals were amplified using long PCR from genomic DNA using a high fidelity hot start DNA polymerase (KapaBiosystems, MA, USA) on a gradient PCR thermocycler (Eppendorf vapo.protect^TM^). PCR primers for single copy amplicons were designed using Primer3 (<http://primer3.ut.ee/> V. 4.0.0) and custom synthesized by Integrated DNA Technologies. Cycling parameters used an initial denaturation of 94^o^C for 4 minutes, followed by 20 second denaturation at 98^o^C for each cycle. The annealing temperature and time (1min/kilobase pair) were optimized for each amplicon. A final extension step at 72^o^C was performed for 10 minutes. These parameters were repeated for 30-35 cycles. Each amplicon were purified using the gel/PCR DNA fragment extraction kit (Geneaid Biotech Ltd., Taiwan) and the amplicon DNA was labeled by nick translation with biotin-dUTP (Roche Diagnostics, ON, Canada) or digoxigenin-dUTP (Roche Diagnostics, ON, Canada). Nick translation reactions were performed at 15^o^C using DNA polymerase I (Roche Diagnostics, ON, Canada), DNase I (Worthington Biochemicals, NJ, USA), 1μg of purified PCR product, and above-mentioned modified dUTPs. To this reaction, a 10μl solution comprised of 100mM dNTPs, 1M Tris-HCl, 1M MgCl_2_, 12.5M 2-Mercaptoethanol (Sigma-Aldrich, ON, Canada), and 20μg/ml bovine serum albumin (Roche Diagnostics, #10711454001) was added. Each nick translation reaction was brought to a final volume of 100ul with nanopure water. Incubation time of the reaction was optimized to obtain nick translated products in a size range of 250-700 base pairs. Biotin or digoxigenin labeled single copy DNA probes were ethanol precipitated and resuspended in 10μl of nanopure water.

***In situ hybridization, detection and imaging*:** Cytogenetic preparations on microscope slides were dehydrated in standard saline solution (2X SSC) for 10 minutes in a 37^o^C water bath, and dehydrated in 80, 90, and 100% ethanol washes (1 minute per wash). Chromosomes were then denatured using ultrapure deionized formamide (70% in 2X SSC) (BioBasic Canada) for 2 minutes, dehydrated in 70 (on ice), 80, 90, and 100%ethanol washes (2 minutes each). An aliquot of labeled probe DNA (150-250ng) was mixed with 10ul of deionized formamide, denatured (10 minutes at 70^o^C) and then mixed with equal volume (10ul) hybridization buffer solution (comprised of 2mg/ml bovine serum albumin, 0.2% SSC, 50% w/v dextran sulfate). The probe mixture was then hybridized to denatured metaphase chromosomes overnight at 37^o^C. Labeled probes were detected with Cy™3 conjugated to IgG fraction monoclonal mouse anti-digoxin (Jackson ImmunoResearch, PA, USA) (diluted 1:200 [1.7mg/ml]) or Alexa Fluor® 488 conjugated to streptavidin (Jackson ImmunoResearch, PA, USA) (diluted 1:500 [1.5mg/ml]) depending on the modified nucleotide incorporated. Post-detection washes were performed in 1X SSC, 1X SSC/0.1% triton-X 100, and 1X SSC, 15 minutes each at room temperature. Cells were stained with 4'-6-Diamidino-2-phenylindole (DAPI; 0.1ug/ml phosphate buffered saline) (EMD, #CA85001-386) for 20 minutes, rinsed in McIlvaine’s buffer (0.1M citric acid, 0.2M disodium phosphate; pH 7.2) for 2 minutes. Microscope slide preparations were mounted in *p-* phenylenediamine antifade. Cells were viewed, analyzed and/or imaged using epifluorescence microscopy or 3-dimensional structured illumination microscopy.
